# *Vital Signs:* Prescription Opioid Pain Reliever Use During Pregnancy — 34 U.S. Jurisdictions, 2019

**DOI:** 10.15585/mmwr.mm6928a1

**Published:** 2020-07-17

**Authors:** Jean Y. Ko, Denise V. D’Angelo, Sarah C. Haight, Brian Morrow, Shanna Cox, Beatriz Salvesen von Essen, Andrea E. Strahan, Leslie Harrison, Heather D. Tevendale, Lee Warner, Charlan D. Kroelinger, Wanda D. Barfield

**Affiliations:** ^1^Division of Reproductive Health, National Center for Chronic Disease Prevention and Health Promotion, CDC; ^2^Division of Overdose Prevention, National Center for Injury Prevention and Control, CDC.

## Abstract

**Background:** Prescription opioid use during pregnancy has been associated with poor outcomes for mothers and infants. Studies using administrative data have estimated that 14%–22% of women filled a prescription for opioids during pregnancy; however, data on self-reported prescription opioid use during pregnancy are limited.

**Methods:** CDC analyzed 2019 data from the Pregnancy Risk Assessment Monitoring System (PRAMS) survey in 32 jurisdictions and maternal and infant health surveys in two additional jurisdictions not participating in PRAMS to estimate self-reported prescription opioid pain reliever (prescription opioid) use during pregnancy overall and by maternal characteristics among women with a recent live birth. This study describes source of prescription opioids, reasons for use, want or need to cut down or stop use, and receipt of health care provider counseling on how use during pregnancy can affect an infant.

**Results:** An estimated 6.6% of respondents reported prescription opioid use during pregnancy. Among these women, 21.2% reported misuse (a source other than a health care provider or a reason for use other than pain), 27.1% indicated wanting or needing to cut down or stop using, and 68.1% received counseling from a provider on how prescription opioid use during pregnancy could affect an infant.

**Conclusions and Implications for Public Health Practice:** Among respondents reporting opioid use during pregnancy, most indicated receiving prescription opioids from a health care provider and using for pain reasons; however, answers from one in five women indicated misuse. Improved screening for opioid misuse and treatment of opioid use disorder in pregnant patients might prevent adverse outcomes. Implementation of public health strategies (e.g., improving state prescription drug monitoring program use and enhancing provider training) can support delivery of evidence-based care for pregnant women.

## Introduction

During 2017–2018, 42.5% of opioid-related overdose deaths among women in the United States involved a prescription opioid (*1*). Long-term use of prescription opioids is associated with increased risk for misuse (i.e., use in larger amounts, higher frequency, longer duration, or for a different reason than that directed by a prescribing physician) (*2*), opioid use disorder, and overdose (*3*,*4*). According to commercial insurance (*5*) and Medicaid (*6*) claims for reimbursement of pharmacy dispensing, an estimated 14%–22% of women filled at least one opioid prescription during pregnancy (*5*,*6*). Opioid use during pregnancy has been associated with poor infant outcomes, such as neonatal opioid withdrawal syndrome (*7*), preterm birth, poor fetal growth, and stillbirth (*8*). PRAMS* and two additional jurisdictions’ maternal and infant health surveys conducted during 2019 were used to describe population-based, self-reported estimates of prescription opioid pain reliever (prescription opioid) use during pregnancy.

## Methods

PRAMS is a jurisdiction-specific and population-based surveillance system designed to monitor self-reported behaviors and experiences before, during, and shortly after pregnancy among women with a live birth in the preceding 2–6 months. Detailed PRAMS methodology is published elsewhere (*9*). Supplementary questions on prescription opioid use during pregnancy were asked in 32 jurisdictions participating in PRAMS and on maternal and infant health surveys in two jurisdictions that do not participate in PRAMS.^†^ Data were weighted to adjust for sample design and nonresponse, representing the total population of women with a live birth in each jurisdiction during an approximately 4-month^§^ or 5-month^¶^ period in 2019.

Women were asked, “During your most recent pregnancy, did you use any of the following prescription pain relievers?” Use of prescription opioid pain relievers (prescription opioids) during pregnancy was indicated by selection of any of the following: hydrocodone, codeine, oxycodone, tramadol, hydromorphone or meperidine, oxymorphone, morphine, or fentanyl.** Women who self-reported use during pregnancy were asked to check all that apply to additional questions describing the prescription opioid source and reasons for use.^††^ Qualitative thematic coding was used to recode “other” written-in text responses into existing and new categories, where possible.^§§^ Remaining responses were retained as “other/undetermined.” Prescription opioid sources were categorized as health care and non–health care provider (based on the responses “I had pain relievers left over from an old prescription,” “friend or family member gave them to me,” or “I got the pain relievers without a prescription some other way”). Reasons for use were categorized as pain and any reason other than pain (based on the responses “to relax or relieve tension or stress,” “to help me with feelings or emotions,” “to help me sleep,” “to feel good or get high,” or “because I was ‘hooked’ or I had to have them”). Misuse was defined as getting opioids from any source other than a health care provider or using for any reason other than pain. Respondents were also asked about their desire to cut down or stop use (“During your most recent pregnancy, did you want or need to cut down or stop using prescription pain relievers?”) and whether they received provider counseling (“At any time during your most recent pregnancy, did a doctor, nurse, or other health care worker talk with you about how using prescription pain relievers during pregnancy could affect a baby?”).

Prevalence of prescription opioid use during pregnancy was estimated overall and by maternal characteristics. Maternal age, race/ethnicity, education, trimester of entry into prenatal care, health insurance at delivery, and number of previous live births were derived from birth certificate data. Self-reported cigarette use during the last 3 months of pregnancy and depression during pregnancy were obtained from the surveys. Among women reporting prescription opioid use during pregnancy, estimates were generated for source, reasons for use, want or need to cut down or stop use, and receipt of health care provider counseling on how use during pregnancy could affect an infant. Prevalence of receipt of health care provider counseling was estimated by maternal characteristics. In addition, the percentage of women who wanted or needed to cut down or stop using was estimated among those who reported misuse as defined in this study and those who did not. Chi-squared tests were used to assess the differential distribution of prescription opioid use during pregnancy and receipt of health care provider counseling by maternal characteristics, as well as the want or need to cut down or stop use by misuse classification. Weighted prevalence estimates and 95% confidence intervals (CIs) were calculated using SUDAAN (version 11.0; RTI International).

## Results

In 2019, among 21,488 respondents, 20,643 (96.1%) provided information regarding prescription opioid use during their most recent pregnancy. Among these women, 1,405 (6.6%) reported prescription opioid use during pregnancy (Table 1). The prevalence of use was statistically different across the following categories: health insurance at delivery, cigarette smoking during the last 3 months of pregnancy, and depression during pregnancy (p<0.05).

**TABLE 1:** Prevalence of self-reported prescription opioid use during pregnancy by maternal characteristics — 34 U.S. jurisdictions, 2019

Characteristic	No. of respondents*	Prevalence of prescription opioid use during pregnancy	
		No.*	%^†^ (95% CI)
**Total**	**20,643**	**1,405**	**6.6 (6.0–7.2)**
**Age group (yrs)**			
≤19	761	56	9.6 (5.8–15.4)
20–24	3,340	246	7.5 (6.0–9.2)
25–34	12,178	822	6.5 (5.7–7.3)
≥35	4,364	281	5.5 (4.6–6.6)
**Race/Ethnicity**			
White, non-Hispanic	9,833	544	5.9 (5.1–6.8)
Black, non-Hispanic	2,798	255	8.6 (6.9–10.5)
Hispanic	5,072	367	7.0 (5.8–8.4)
Other, non-Hispanic**^§^**	2,665	218	6.6 (5.3–8.2)
**Education level (yrs)**			
<12	2,292	203	8.4 (6.4–11.0)
12	4,568	369	7.1 (6.0–8.4)
>12	13,415	805	6.1 (5.4–6.9)
**Trimester of entry into prenatal care**			
First	16,241	1,072	6.2 (5.6–6.9)
Second, third, or none	3,124	205	6.3 (4.9–7.9)
**Health insurance at delivery** ^¶^			
Private**	10,653	591	5.2 (4.6–6.0)
Medicaid	8,317	712	8.5 (7.5–9.7)
Other^††^ or none	1,068	59	4.4 (2.9–6.5)
**No. of previous live births**			
None	7,982	504	6.3 (5.4–7.3)
One or more	12,508	885	6.7 (6.0–7.5)
**Smoked cigarettes during last 3 mos of pregnancy** ^¶^			
Yes	1,279	192	16.2 (12.7–20.4)
No	19,227	1,200	5.9 (5.4–6.5)
**Depression during pregnancy** ^¶^ **^,^** ^§§^			
Yes	2,432	295	13.1 (10.7–15.8)
No	12,319	730	5.4 (4.8–6.1)

**Abbreviation:** CI = confidence interval.

* Unweighted sample size.

^†^ Weighted prevalence (expressed as a percentage).

^§^ Includes Asian, American Indian, Alaska Native, Native Hawaiian, Pacific Islander, and mixed race/ethnicity.

^¶^ Indicates chi-squared test p<0.05.

** Includes Civilian Health and Medical Program of the Department of Uniformed Services and TRICARE.

^††^ Includes Children’s Health Insurance Program and other government programs.

^§§^ California data not available.

Among women who used prescription opioids, 91.3% reported receiving the opioids from a health care provider, 8.9% from a source other than a health care provider (e.g., friend or family member), and 4.3% from other/undetermined sources (Table 2). Specifically, 55.4% of women reported receiving opioids from an obstetrician-gynecologist, midwife, or prenatal care provider and 26.0% from an emergency department doctor. The two most commonly reported non–health care provider sources were having pain relievers left over from an old prescription (5.4%) and obtaining the pain relievers without a prescription some other way (3.0%).

**TABLE 2:** Sources of prescription opioids and reasons for use among respondents reporting use during pregnancy (N = 1,405) — 34 U.S. jurisdictions, 2019

Sources of opioids/Reasons for use	No.*	Prevalence %^†^ (95% CI)
**Source of prescription opioid**	1,335	—
Any health care provider source	1,233	91.3 (88.0–93.7)
Ob/gyn, midwife, or prenatal care provider	787	55.4 (50.4–60.2)
Family doctor or primary care provider	203	14.9 (11.6–18.9)
Dentist or oral health care provider	139	12.8 (9.7–16.8)
Doctor in the emergency department	352	26.0 (22.0–30.4)
Other health care provider	50	2.7 (1.6–4.7)
Any non-health care provider source	132	8.9 (6.7–11.8)
Pain relievers left over from old prescription	74	5.4 (3.6–7.9)
Friend or family member	36	1.9 (1.2–3.1)
Some other way without a prescription	52	3.0 (1.9–4.7)
Other/Undetermined	53	4.3 (2.6–7.1)
**Reason for prescription opioid use**	1,303	—
Any pain reason	1,131	88.8 (85.9–91.2)
To relieve pain from an injury, condition, or surgery before pregnancy	264	22.2 (18.3–26.7)
To relieve pain from an injury, condition, or surgery during pregnancy	807	63.8 (59.1–68.2)
To relieve pain from an injury, condition, or surgery unstated time frame	183	11.7 (9.1–14.9)
Any reason other than pain	204	14.4 (11.2–18.4)
To relax or relieve tension or stress	118	7.7 (5.5–10.8)
To help with feelings or emotions	45	3.7 (2.0–6.8)
To help sleep	115	7.9 (5.4–11.3)
To feel good or get high	23	1.1 (0.6–2.0)
Because ”hooked” or had to use	32	2.4 (1.2–4.8)
Other/Undetermined	88	4.9 (3.7–6.6)
**Any misuse (non–health care provider source or reasons other than pain)**	277	21.2 (17.3–25.6)

**Abbreviations:** CI = confidence interval; ob/gyn = obstetrician/gynecologist.

* Unweighted sample size.

^†^ Weighted prevalence (expressed as a percentage) will not sum to 100% because of questions that asked respondents to check all answers that applied.

Among women who used prescription opioids, 88.8% reported using the opioids for pain reasons, 14.4% for reasons other than pain, and 4.9% for other/undetermined reasons. In particular, prescription opioids were used to relieve pain from an injury, condition, or surgery that occurred before (22.2%) or during (63.8%) pregnancy or during an unstated time frame (11.7%). Commonly reported reasons for use other than pain were to help sleep (7.9%) and relieve tension or stress (7.7%).

Overall, 21.2% of women who used prescription opioids during pregnancy reported misuse; 4.0% reported both a non–health care provider source and use for reasons other than pain. Among women who used prescription opioids during pregnancy, 27.1% indicated wanting or needing to cut down or stop using (Figure). Among women who used prescription opioids during pregnancy, a higher proportion of women with misuse (36.5%) indicated wanting or needing to cut down or stop using, compared with women without misuse (24.5%) (p<0.05).

**FIGURE:**
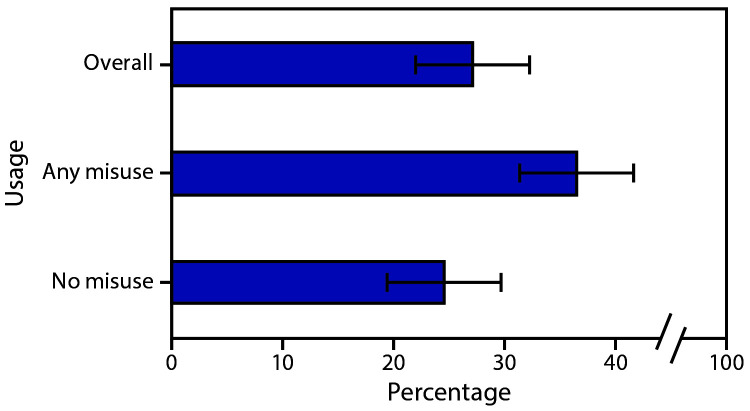
Percentage of women reporting desire to cut down or stop using prescription opioids among respondents reporting use*^,†^ during pregnancy (N = 1,405) — 34 U.S. jurisdictions, 2019 * “Any misuse” includes report of any sources other than a health care provider (including “I had pain relievers left over from an old prescription,” “friend or family member gave them to me,” “I got the pain relievers without a prescription some other way” or “other”) or reasons other than pain (including “to relax or relieve tension or stress,” “to help me with feelings or emotions,” “to help me sleep,” “to feel good or get high,” “because I was ‘hooked’ or I had to have them” or “other”). ^†^ “No misuse” indicates that respondents reported only health care provider sources and pain reasons.

Among women with prescription opioid use during pregnancy, 68.1% reported that a health care provider counseled them about the effect of use on an infant (Table 3). The prevalence of receiving counseling did not vary by most maternal characteristics assessed except that a lower proportion of women with no previous live births received counseling than did those with one or more previous births (62.0% versus 71.6%; p<0.05).

**TABLE 3:** Prevalence of provider counseling on how using prescription opioids during pregnancy could affect a baby among women who self-reported prescription opioid use (N = 1,373) — 34 U.S. jurisdictions, 2019

Characteristic	Total	Prevalence of provider counseling	
	No.*	No.*	%^†^ (95% CI)
**Total**	**1,373**	**887**	**68.1 (63.8–72.1)**
**Age group (yrs)**			
≤19	55	34	62.2 (36.9–82.2)^§^
20–24	240	153	60.7 (49.6–70.8)
25–34	807	524	71.1 (65.7–75.9)
≥35	271	176	69.0 (60.1–76.6)
**Race/Ethnicity**			
White, non-Hispanic	528	338	65.2 (58.2–71.6)
Black, non-Hispanic	254	167	70.1 (60.1–78.4)
Hispanic	357	224	72.4 (64.9–78.9)
Other, non-Hispanic^¶^	214	143	67.4 (56.2–76.9)
**Education level (yrs)**			
<12	192	118	59.6 (45.1–72.6)
12	361	234	68.8 (60.8–75.9)
>12	793	515	69.4 (63.9–74.4)
**Trimester of entry into prenatal care**			
First	1,052	688	70.2 (65.4–74.6)
Second, third, or none	200	125	61.6 (49.9–72.2)
**Health insurance at delivery**			
Private**	582	379	71.6 (65.5–77.0)
Medicaid	694	455	67.6 (61.1–73.5)
Other^††^ or none	54	32	57.1 (36.7–75.3)^§^
**No. of previous live births** ^§§^			
None	494	308	62.0 (54.3–69.2)
One or more	863	570	71.6 (66.5–76.2)
**Smoked cigarettes during last 3 mos of pregnancy**			
Yes	185	107	64.0 (51.4–75.0)
No	1,175	770	68.7 (64.0–73.0)
**Depression during pregnancy** ^¶¶^			
Yes	289	195	76.0 (67.2–83.1)
No	709	457	65.9 (59.9–71.4)

**Abbreviation:** CI = confidence interval.

* Unweighted sample size.

^†^ Weighted prevalence (expressed as a percentage).

^§^ Denominator is less than <60, so estimate may be unstable.

^¶^ Includes Asian, American Indian, Alaska Native, Native Hawaiian, Pacific Islander, and mixed race/ethnicity.

**Includes Civilian Health and Medical Program of the Department of Uniformed Services and TRICARE.

^††^ Includes Children’s Health Insurance Program and other government programs.

^§§^ Indicates chi-squared test p<0.05.

^¶¶^ California data not available.

## Discussion

In this population-based sample of women with recent live births in 34 jurisdictions, one in 15 (6.6%) respondents self-reported using prescription opioid pain relievers during pregnancy. This observed prevalence of use during pregnancy in 2019 is lower than estimates of prescription opioid fills from administrative data (e.g., insurance claims) in previous years (*5*,*6*), which do not necessarily correlate with use. Higher use of prescription opioids among women who reported smoking cigarettes or had depression during pregnancy are consistent with findings from studies analyzing administrative Medicaid data (*7*).

In this study, an estimated one in five women using prescription opioids during pregnancy indicated misuse. In addition, more than one in four (27.1%) women with prescription opioid use indicated wanting or needing to reduce or stop their use, potentially because of concerns about the effect of medication on their infant, possible opioid dependence, or opioid use disorder. Among women reporting prescription opioid use, nearly one in three (31.9%) reported not receiving provider counseling on the effects of prescription opioid use on an infant.

Clinical guidance addresses opioid prescribing and tapering during pregnancy, the risks to the mother and infant, and screening and treatment for opioid dependence and opioid use disorder (*3*,*10*). CDC and the American College of Obstetricians and Gynecologists (ACOG) recommend that clinicians and patients discuss and carefully weigh risks and benefits when considering initiation of opioid therapy for chronic pain during pregnancy (*3*,*10*). Opioids, if indicated, should be prescribed only after consideration of alternative pain management therapies (*3*,*10*). Risk for physiologic dependence and possibility of an infant developing neonatal opioid withdrawal syndrome should be discussed (*10*). Clinicians caring for pregnant women are advised to perform verbal screening to identify and address substance use, misuse, and substance use disorders (*10*,*11*). Co-occurring use of other substances (e.g., tobacco) and mental health conditions are more common among pregnant women who are prescribed or misusing prescription opioids than among those who are not (*7*,*12*). Recommended screening and, if applicable, treatment and referral for depression, history of trauma, posttraumatic stress disorder, and anxiety should occur (*10*). Because of the possible risk for spontaneous abortion and premature labor associated with opioid withdrawal (*10*), clinicians are encouraged to consult with other health care providers as necessary if considering tapering opioids during pregnancy (*3*). Medications for opioid use disorder, including buprenorphine or methadone, are recommended because of their association with improved maternal outcomes (*3*,*10*,*13*). Collaboration between obstetric and neonatal providers is important to diagnose, evaluate, and treat neonatal opioid withdrawal syndrome because it can result from medically indicated opioid prescription use, medication for opioid use disorder, or illicit opioid use (*3*,*10*).

Effective public health strategies to support the implementation of evidence-based guidelines might include improving state prescription drug monitoring program use (*14*), provider training (*15*), multidisciplinary state learning communities (*16*), quality improvement collaboratives (*17*), and consumer awareness (*18*). For example, some state perinatal quality collaboratives are implementing the Alliance for Innovation on Maternal Health program’s patient safety obstetric care bundle for pregnant and postpartum women with opioid use disorder to implement protocols for screening and referral to treatment (*16*,*19*).

The findings in this report are subject to at least five limitations. First, these population-based data are only generalizable to women with a recent live birth in the 34 jurisdictions included in this report. Because of the need to provide data on the opioid crisis among pregnant women, a response rate threshold was not required for jurisdictions to be included in the analyses. This might further affect generalizability because 13 jurisdictions fell below the current PRAMS threshold of 55% (*9*). Second, prescription opioid use was self-reported and might be underestimated because of stigma and legal implications.^¶¶^ Third, question misinterpretation by respondents is possible. For example, <1% indicated no source or reason for use except for a written-in response regarding use during labor and delivery, even though the initial prompt asked women to not include pain relievers used during labor and delivery. Fourth, not all available misuse indicators (e.g., use for longer time than prescribed) were assessed. Finally, the opioid supplement questions do not reflect current diagnostic criteria and cannot be used to estimate the prevalence of opioid use disorder (*20*).

Opioid prescribing consistent with clinical practice guidelines can ensure that patients, particularly those who are pregnant, have access to safer, more effective chronic pain treatment and reduce the number of persons at risk for opioid misuse, opioid use disorder, and overdose. Implementation of public health strategies can complement these efforts to improve the health of mothers and infants. The PRAMS surveillance system can be used to identify opportunities for providers, health systems, and jurisdictions to better support pregnant and postpartum women and their families.

**Summary**

**What is already known about this topic?**

Data on self-reported prescription opioid use during pregnancy are limited.

**What is added by this report?**

Analysis of 2019 survey data found that 6.6% of women reported prescription opioid use during pregnancy. Among these women, 21.2% reported misuse (a source other than a health care provider or a reason for use other than pain), 27.1% wanted or needed to cut down or stop using, and 31.9% reported not receiving provider counseling about how use could affect an infant.

**What are the implications for public health practice?**

Obstetric providers should discuss risks and benefits of opioid therapy for chronic pain during pregnancy, screen all pregnant women for substance use, misuse, and use disorders, including those involving prescription opioids, and provide referral and treatment, as indicated.

## Footnotes

* PRAMS currently requires that jurisdictions meet a response rate threshold of 55% for publication. However, because of the critical need to report surveillance data related to the opioid crisis, a response rate threshold was not used to determine inclusion in the analysis. Therefore, data in this report are from all PRAMS jurisdictions participating in the opioid supplement (response rate noted): Alabama (57.6%), Arizona (41.9%), Colorado (59.7%), Connecticut (52.6%), District of Columbia (48.4%), Florida (46.1%), Georgia (53.6%), Illinois (62.6%), Indiana (46.4%), Iowa (56.7%), Kansas (66.0%), Kentucky (61.5%), Louisiana (55.9%), Maryland (47.8%), Massachusetts (61.2%), Missouri (56.5%), Nevada (43.5%), New Hampshire (51.0%), New York (51.4%), North Dakota (57.3%), Oregon (69.6%), Pennsylvania (55.6%), Puerto Rico (81.1%), Rhode Island (57.1%), South Carolina (38.3%), South Dakota (69.4%), Tennessee (55.0%), Utah (71.9%), Vermont (61.6%), Washington (60.6%), West Virginia (42.7%), and Wyoming (56.3%)

† California (response rate: 59.3%) and Ohio (response rate: 34.2%)

§ California collected data during a 4-month period; the weight was adjusted for this analysis to represent mothers giving birth in this approximately 4-month data collection period in 2019.

¶ For PRAMS jurisdictions and Ohio, 5 months of data were weighted to represent women having a live birth during approximately 5 months in 2019.

** Survey options for prescription opioids included “Hydrocodone (like Vicodin, Norco, or Lortab)”; “Codeine (like Tylenol #3 or #4, not regular Tylenol”); “Oxycodone (like Percocet, Percodan, OxyContin, or Roxicodone)”; “Tramadol (like Ultram or Ultracet); Hydromorphone or meperidine (like Demerol, Exalgo, or Dilaudid)”; “Oxymorphone (like Opana)”; “Morphine (like MS Contin, Avinza, or Kadian)”; or “Fentanyl (like Duragesic, Fentora, or Actiq).”

†† Respondents were asked, “Where did you get the prescription pain relievers that you used during your most recent pregnancy?” (answer options included “OB-GYN, midwife, or prenatal care provider,” “family doctor or primary care provider,” “dentist or oral health care provider,” “doctor in the emergency room,” “I had pain relievers left over from an old prescription,” “friend or family member gave them to me,” “I got the pain relievers without a prescription some other way,” and “other.”) and “What were your reasons for using prescription pain relievers during your most recent pregnancy?” (answer options included “to relieve pain from an injury, condition, or surgery I had before pregnancy,” “to relieve pain from an injury, condition, or surgery that happened during my pregnancy,” “to relax or relieve tension or stress,” “to help me with my feelings or emotions,” “to help me sleep,” “to feel good or get high,” “because I was ‘hooked’ or I had to have them,” and “other”).

§§ Written-in responses regarding receiving prescription opioids from a health care provider (e.g., gastrointestinal provider, specialist, or surgeon) not listed as an option were coded as “other health care provider.” Written-in responses regarding using prescription opioids to relieve pain from a medical condition, but with no indicated timeframe, were coded as “to relieve pain from an injury, condition, or surgery that occurred during an unstated timeframe.” Written-in responses indicating use not occurring during pregnancy (e.g., “only during labor and delivery,” “did not use during pregnancy”) were retained as “other” if no other source or reason was indicated.

¶¶ https://www.guttmacher.org/state-policy/explore/substance-use-during-pregnancy.
